# Oribatid mite communities in mountain scree: stable isotopes (^15^N, ^13^C) reveal three trophic levels of exclusively sexual species

**DOI:** 10.1007/s10493-021-00597-4

**Published:** 2021-03-01

**Authors:** Ioana Nae, Augustin Nae, Stefan Scheu, Mark Maraun

**Affiliations:** 1 Emil Racoviță Institute of Speleology of Romanian Academy, 13 Septembrie Road, No. 13, 050711 Bucharest, Romania; 2 J.F. Blumenbach Institute of Zoology and Anthropology, Animal Ecology, University of Gӧttingen, Untere Karspüle 2, 37073 Gӧttingen, Germany

**Keywords:** Stable isotopes, Oribatid mites, Trophic level, Carpathians, Romania

## Abstract

Mountain scree habitats are intermediate habitats between the base of the soil and the bedrock. They are composed of a network of small cracks and voids, and are commonly situated at the lower levels of scree slopes. Their environment is defined by empty spaces inside the scree, the absence of light and photoperiod, low temperature, and resource poor conditions. Soil arthropod communities, their trophic structure as well as their use of basal resources in mountain scree are little studied despite the fact that they are important components of these systems. Here, we investigate stable isotope ratios (^15^N/^14^N, ^13^C/^12^C) of oribatid mites (Oribatida, Acari) to understand their trophic niches and their variation with depth (50 and 75 cm) at two mountain scree sites (Cerdacul Stanciului, Marele Grohotis) in the Romanian Carpathians. Further, we used existing data to investigate the reproductive mode of the species in that habitat, as this may be related to resource availability. We hypothesized that trophic niches of oribatid mites will not differ between the two mountain scree regions but will be affected by depth. We furthermore hypothesized that due to the resource poor conditions oribatid mite species will span a narrow range of trophic levels, and that species are sexual rather than parthenogenetic. Our results showed that (1) oribatid mite trophic structure only slightly differed between the two sites indicating that the trophic ecology of oribatid mites in scree habitats is consistent and predictable, (2) oribatid mite trophic structure did not differ between the two studied soil depths indicating that the structure and availability of resources that were used by oribatid mites in deeper scree habitats varies little with depth, (3) oribatid mite species spanned only three trophic levels indicating that the habitat is rather resource poor, and (4) that all studied oribatid mite species were sexual supporting the view that resource poor conditions favour sexual reproduction.

## Introduction

Mountain scree habitats are commonly found in scree slopes, where soil has not filled in the spaces. Scree represents an intermediate habitat between soil and bedrock. It is composed of a network of small cracks and voids, and usually is situated at the lower level of scree slopes (Juberthie et al. 1980; Juberthie 1983; Nitzu et al. 2014). Mountain scree habitats are characterised by little light and photoperiodicity, contrasting caves where at the entrance the presence of light allows vegetation development. Mountain scree habitats are characterized by very low availability of resources; however, the availability of resources may differ between mountain scree regions and it may also decline with depth (Culver and Pipan 2019). Resources enter this habitat by (1) water (hydrochoric transportation), (2) gravity (gravitational transportation), and (3) active animal migrations from the surface or from the deep hypogean domain (biochoric transportation) (Mammola et al. 2016). The limited availability of resources makes this habitat an ideal study site for understanding the trophic ecology and the use of basal resources of soil animals such as oribatid mites under resource poor conditions (Mumladze et al. 2015).

Oribatid mites (Oribatida, Acari) are an important component of the arthropod community in virtually all terrestrial ecosystems (Maraun and Scheu 2000). They feed on decomposing plant material and fungi, but also on lichens and living or dead animals (Walter and Proctor 1999; Schneider et al. 2004), representing the full decomposer community. Oribatid mites also are an ideal group to investigate the factors affecting soil animal community structure, including both density-independent (Lindberg and Bengtsson 2005; Badejo and Akinwole 2006; Bluhm et al. 2016) and density-dependent factors (Fischer et al. 2010; Caruso et al. 2013). Oribatid mite diversity varies among habitats (Schatz and Behan-Pelletier 2008), although the factors responsible for their variation in space are little understood (Maraun and Scheu 2000; Caruso et al. 2019). Oribatid mite communities of subterranean habitats such as mountain scree have been studied intensively (Skubała et al. 2013; Jiménez-Valverde et al. 2015; Nae and Băncilă 2017), but their trophic structure is little known.

Around 10% of oribatid mite species reproduce by parthenogenesis, i.e., thelytoky, which is much higher than in other invertebrate and vertebrate taxa except in bdelloid rotifers (Fischer et al. 2010). In some ecosystems, even more than 90% of the individuals of oribatid mites in soil reproduce via parthenogenesis (Norton and Palmer 1991; Maraun et al. 2003, 2012). By contrast, in caves and scree habitats both the number of sexual oribatid mite species and individuals are high, whereas their densities are low (Maraun et al. 2019). Presumably, resource limitation triggers the abundance and frequency of sexual taxa, especially in soil animals (Scheu and Drossel 2007).

Increasingly, trophic niches of animals are being analysed measuring natural variations in stable isotope ratios of nitrogen (^15^N/^14^N) and carbon (^13^C/^12^C). The method provides integrative insight into the trophic position of consumers and also into the use of basal resources in soil animal communities (Scheu and Falca 2000; Tiunov 2007; Maraun et al. 2011; Potapov et al. 2019). Consumers are enriched in ^15^N by an average of 3.4 δ units relative to their food source (DeNiro and Epstein 1981; Post 2002; Martinez del Rio et al. 2009). By contrast, fractionation of ^13^C is lower, averaging ~ 0.4 δ units per trophic level (Post 2002; Martinez del Rio et al. 2009), and therefore is of little use to determine the trophic structure of communities (Ponsard and Arditi 2000); however, in particular in soil it allows to trace the use of basal resources of consumers (Albers et al. 2006; Pollierer et al. 2009; Melguizo-Ruiz et al. 2017; Potapov et al. 2019).

The present study investigates for the first time the trophic structure of oribatid mite species in mountain scree using stable isotopes (^13^C, ^15^N). Additionally, we use existing data to investigate the reproductive mode in this particular subterranean habitat. We hypothesized that (1) the trophic niches of oribatid mites differ little in space, i.e., between scree habitats as environmental conditions in scree habitats are similar. We further hypothesized that (2) the trophic structure of oribatid mite communities will change with soil depth with species lower in the food web being more frequent at deeper layers of scree habitats. We also hypothesized that (3) the number of trophic levels in this resource poor habitat will be lower than reported from soils of forests and meadows due to resource scarcity. Finally, we hypothesized that (4) the reproductive mode of oribatid mites in mountain scree is mainly sexual, as resource poor habitats are assumed to be dominated by sexual species.

## Material and methods

### Study sites

**Fig. 1:**
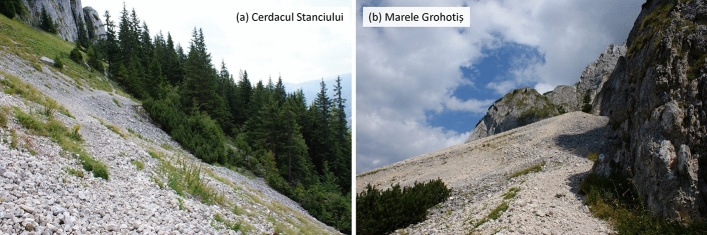
The two mountain scree habitats studied at Cerdacul Stanciului (**a**) and Marele Grohotiș (**b**)

Oribatid mites were sampled in the framework of a long-term study on invertebrate communities in scree habitats and caves in Piatra Craiului National Park, Southern Carpathians, one of the most important karst areas of Romania (Nitzu et al. 2014). Piatra Craiului Massif is a 20 km^2^ limestone ridge, where more than 500 caves were identified and diverse types of talus and scree slopes, both covered and open, are present (Culver and Pipan 2009). Two types of scree slopes were selected, (1) Cerdacul Stanciului, a mobile limestone scree situated near Stanciului Cave and (2) Marele Grohotis, the largest mobile nude limestone scree accumulation from Piatra Craiului Massif (Fig. 1).

Cerdacul Stanciului and Marele Grohotis are sub-alpine habitats located at 1650 and 1580 m, respectively, and were classified as "calcareous and calcashist screes of the montane to alpine level – Thlaspietea rotundifolii" (Doniță et al. 2005) and are listed in the 8210 habitat types following the Natura 2000 habitat classification. Doniță et al. (2005) included both sites in R6109 type: South-Eastern Carpathian communities of semi-mobile and mobile screes. Mean temperature between April and November 2008 at both scree sites is ca. 16 °C at 50 cm and ca. 10 °C at 75 cm depth (for details see Nae and Băncilă 2017).

Piatra Craiului Massif is a limestone ridge and this type of rock represents 39.5% of the ridge, which favours water infiltration. Therefore, the circulation of underground water plays an important role in the denudation of the relief. Most of the precipitation is infiltrating the rock, resulting in humidity deficiency on the limestone slopes and preventing soil or vegetation development (Constantinescu 2009). This process favours gelifraction, a process that produces rock torrents (which forms through rolling and collapsing limestone), the characteristic geological forms found in Piatra Craiului.

### Sampling, extraction and determination

Oribatid mites included in this study were collected using drillings, a special type of pitfall traps adapted for screes (López and Oromi 2010). They consist of PVC tubes, with a pitfall trap inserted at the base. The upper part of the drilling was covered with a plastic lid to prevent debris and rocks from falling inside and the tube had holes drilled at the base, so only animals from that level could enter the trap. We assumed this way of sampling to collect the whole soil oribatid mite community; however, this needs confirmation using other sampling techniques such as heat extraction. Each trap was filled by half with 70% ethanol and an attractant (fermented cheese; Jordana et al. 2020), and was emptied once a month from April to November in 2008 and 2009. The mountain scree habitat was sampled at two depths, 50 and 75 cm.

Oribatid mites were sorted under the stereo microscope and identified to species level with a microscope (Olympus CH2) using the keys of van der Hammen (1952, 1959), Bernini (1978), Pérez-Iñigo (1993, 1997) and Weigmann (2006). The systematic ranking of the species was done after Subías (2004). After identification, the material was preserved in 70% ethanol and stored until stable isotope analysis. The sexing of the species was done based on the list in the Appendix of Maraun et al. (2019).

### Stable isotope analysis

Stable isotope ratios of abundant oribatid mite species of the study sites were analysed (see species list in Table 1). Between 1 and 100 individuals were bulked per sample ranging between 1.17 and 10.93 mg of animal tissue.

**Table 1:** Names and number of occurrences of oribatid mite species at the two study sites Marele Grohotiș and Cerdacul Stanciului at 50 and 75 cm soil depth

Species	Marele Grohotiș		Cerdacul Stanciului	
	50 cm	75 cm	50 cm	75 cm
*Ceratoppia bipilis *(Hermann)	50	30	10	10
*Oribatella *(*O.*)* foliata* Krivolutsky	20	3	10	3
*Chamobates cuspidatus* (Michael)	14	18	7	0
*Euzetes globulus* (Nicolet)	12	0	0	0
*Oribatella *(*O.*)* quadricornuta* (Michael)	10	0	0	2
*Parachipteria willmanni* (van der Hammen)	8	4	0	0
*Oribatella *(*O.*)* calcarata* (Koch)	2	3	2	0
*Oribatula *(*O.*)* tibialis* (Nicolet)	4	1	0	0
*Hermannia *(*H.*)* gibba* (Koch)	4	4	0	0
*Eupelops torulosus* (Koch)	3	4	0	0
*Liacarus *(*L.*)* coracinus* (Koch)	3	2	0	0
*Carabodes *(*C.*)* labyrinthicus* (Michael)	0	3	0	0
*Carabodes *(*C.*)* marginatus* (Michael)	3	0	0	0
*Carabodes *(*C.*)* areolatus* Berlese	1	0	0	0
*Eupelops acromios* (Hermann)	2	1	0	0
*Xenillus *(*X.*)* tegeocranus* (Hermann)	1	4	0	0
*Liacarus *(*Dorycranosus*)* acutus* Pschorn-Walcher	4	0	0	0

Food resources of oribatid mite species in mountain scree originate from the epigean environment (Mammola et al. 2016), and therefore we collected surface soil samples at both study sites using a small spade. Both sampling sites have a similar vegetation cover and type of soil. Additionally, we sampled potential food resources of oribatid mites from the study sites, namely roots, mosses and lichens. All samples were dried at 100 °C for 24 h in a desiccator, grounded with mortar and pestle, and subsequently measured for ^15^N and ^13^C signatures.

^15^N/^14^N and ^13^C/^12^C ratios of oribatid mites and surface samples were determined by a coupled system of an elemental analyser (NA1110, Carbo Erba, Milan) and a mass spectrometer (MAT Delta Plus, Finnigan). Isotope signatures are reported using the δ notation with δ^15^N or δ^13^C (‰) = (R_sample_ – R_standard_)/R_standard_ × 1000, with R_sample_ and R_standard_ representing the ^15^N/^14^N and ^13^C/^12^C ratios of the sample and the standard, respectively. Nitrogen in atmospheric air was used as primary standard for ^15^N, and acetanilide was used for internal calibration.

### Statistical analysis

Before statistical analysis, stable isotope data of oribatid mite species and their potential food resources (soil, mosses and lichens) from the two study sites ‘Cerdacul Stanciului’ and ‘Marele Grohotis’ were calibrated to the stable isotope signature of roots used as baseline. The calibrated data were analysed using Discriminant Function Analysis (DFA) with the grouping variable ‘site’ with two levels (‘Cerdacul Stanciului’ and ‘Marele Grohotis’), and the ^15^N and ^13^C values of oribatid mites as dependent variables. Subsequently, we used ANOVA to separately analyse ^15^N and ^13^C values of the oribatid mite species that occurred at both sites.

Only two oribatid mite species occurred at both sites in sufficient numbers (i.e., at least two replicates per site) for statistical comparison, namely *Oribatella foliata* and *Ceratoppia bipilis*. We investigated if their stable isotope signatures differed between sites. We performed a Discriminant Function Analysis (DFA) with the grouping variable ‘sites’ with two levels (‘Cerdacul Stanciului’ and ‘Marele Grohotis’), and the two dependent factors ‘^15^N’ and ‘^13^C’. As the analysis was only significant for *C. bipilis* (for details see below) we subsequently tested (using ANOVA) which of the two factors (‘^15^N’ and ‘^13^C’) was responsible for the differences. Second, we performed another DFA with the grouping variable ‘depth’ with two levels (50 and 75 cm) and the two independent factors ‘^15^N’ and ‘^13^C’. All statistical analyses were carried out using Statistica v.13.5.0 (Tibco Statistica, Palo Alto, CA, USA; https://docs.tibco.com/products/tibco-statistica-13-5-0).

## Results

Twelve oribatid mite species exclusively occurred at the Marele Grohotis site (Table 1), whereas no oribatid mite species occurred exclusively at the Cerdacul Stanciului site; five oribatid mite species occurred at both sites (*Ceratoppia bipilis*, *Chamobates cuspidatus*, *Oribatella calcarata*, *Oribatella foliata* and *Oribatella quadricornuta*); however, only two species (*C. bipilis* and *O. foliata*) occurred at both sites in sufficient numbers for statistical analyses.

### Variations between study sites

Stable isotope signatures (root calibrated data) of the oribatid mite communities from ‘Cerdacul Stanciului’ and ‘Marele Grohotis’ were significantly different (DFA: Wilks’ λ = 0.81; approx. F_2,53_ = 6.01, p = 0.004) which was due to differences in ^13^C (ANOVA: F_1,54_ = 10.60, p = 0.002) but not in ^15^N values (F_1,54_ = 1.80, p = 0.18). However, only two species (*O. foliata* and *C. bipilis*) occurred in sufficient numbers to allow at least two stable isotope measurements per site. ^15^N and ^13^C values of *O. foliata* did not differ significantly between ‘Cerdacul Stanciului’ and ‘Marele Grohotis’ (DFA: Wilks’ λ = 0.14; approx. F_2,2_ = 5.94, p = 0.14), whereas those of *C. bipilis* differed significantly between the two sites (DFA: Wilks’ λ = 0.13; approx. F_2,7_ = 21.93, p < 0.001), which was due to differences in ^13^C (ANOVA: F_1,8_ = 40.9, p < 0.001) but not in ^15^N values (F_1,8_ = 0.50, p = 0.50).

### Variations with soil depth

The depth where the samples were taken from (50 and 75 cm) did not significantly affect the stable isotope signatures of oribatid mite species (root calibrated data) at the two study sites (DFA: Wilks’ λ = 0.97; approx. F_2,53_ = 0.79, p = 0.46).

### Number of trophic levels

**Fig. 2:**
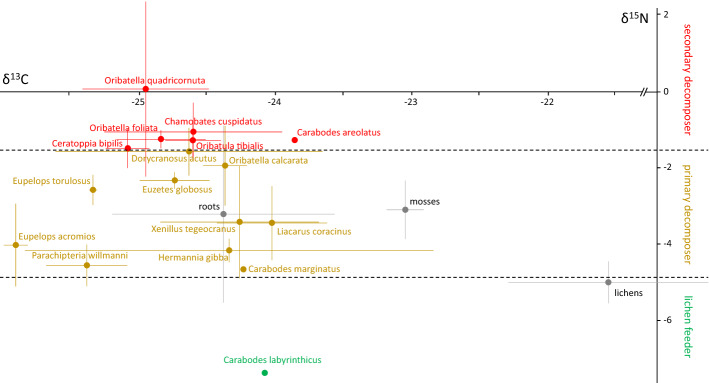
Stable isotope signatures (δ^13^C [‰] and δ^15^N [‰]) (means ± SD) of oribatid mite species from the studied mountain scree habitats in the Romanian Carpathians (data pooled for the two study sites, Cerdacul Stanciului and Marele Grohotis, and the two sampling depths, 50 and 75 cm). Signatures of roots, mosses and lichens are given for comparison

^15^N data of oribatid mite species pooled for study sites indicated that they comprised three trophic levels (Fig. 2). The lowest trophic level (lichen feeders) included only one species, *Carabodes labyrinthicus*. Ten species were grouped as primary decomposers including *C. marginatus*, *D. acutus*, *E. globosus*, *E. acromios*, *E. torulosus*, *H. gibba*, *L. coracinus*, *O. calcarata*, *P. willmanni* and *X. tegeocranus*. Six species were grouped as secondary decomposers/fungal feeders including *C. cuspidatus*, *C. bipilis*, *C. areolatus*, *O. foliata*, *O. quadricornuta* and *O. tibialis*. Species of the same genus often were ascribed to different trophic levels, e.g., in the three species of the genus *Carabodes*, with *C. marginatus* grouped as primary decomposer, *C. areolatus* as secondary decomposer and *C. labyrinthicus* as lichen feeder. Similarly, in *Oribatella* species, *O. calcarata* was grouped as primary decomposer, whereas *O. quadricornuta* was grouped as secondary decomposer. By contrast, both *Eupelops* species (*E. torulosus* and *E. acromios*) were grouped as primary decomposers.

### Reproductive mode

All species recorded at the two study sites were sexual suggesting that parthenogenetic species do not exist or are very rare.

## Discussion

### Trophic structure

Findings of our study indicated that stable isotope signatures between oribatid mite communities from the two mountain scree sites (Cerdacul Stanciului, Marele Grohotis) differed only little, and that the (limited) differences between the two communities likely were due to different species occurring at the two sites rather than differences in trophic niches between the same species. In fact, the isotope signatures of the species that occurred at both sites in sufficient numbers to allow stable isotope analysis were similar. Only one species, *C. bipilis*, had higher ^13^C signatures in Cerdacul Stanciului than in Marele Grohotis. This suggests that the food resources used by oribatid mite communities at the two sites are similar.

The trophic structure of oribatid mite communities at the two study sites was not significantly affected by the depth of the scree they were sampled (50 and 75 cm). This indicates that the composition of food resources that are consumed by oribatid mite species changes little with depth, at least in deep scree habitats. Presumably, food resources originate in large from the plants (including cryptogams) growing at the surface or from soil covering scree slopes. Little variation in trophic niches with depth in oribatid mites in scree habitats is in agreement with other studies where stable isotope values of oribatid mites also have been shown to vary little with soil depth (Scheu and Falca 2000; Potapov et al. 2019).

Oribatid mite communities at the two studied mountain scree sites spanned three trophic levels and included lichen feeders, primary decomposers and secondary decomposers. However, lichen feeders were only represented by one species (which only occurred at low density at one site, Marele Grohotis); based on detritus as basal resource the communities only included two trophic levels, i.e., primary and secondary decomposers. Compared to other habitats this is rather low; for oribatid mites in temperate and tropical forest, in meadows and in salt marshes the number of trophic levels (as indicated by δ^15^N values) typically is four (Illig et al. 2005; Fischer et al. 2010; Perdomo et al. 2012; Magilton et al. 2019). Only two studies on oribatid mites in dead wood (Bluhm et al. 2015) and in sporocarps of *Fomitopsis* (Maraun et al. 2014) reported only three trophic levels. This indicates that the resource conditions in mountain scree are rather poor restricting energy supply of higher trophic levels (Brown et al. 2004).

### Reproductive mode

All oribatid mite species recorded were reproducing sexually, whereas parthenogenetic oribatid mite taxa dominating in many terrestrial habitats, such as Nothrina, Brachychthoniidae, Suctobelbidae and species of the genus *Tectocepheus* (Maraun and Scheu 2000), were missing. As indicated by low density and few trophic levels, mountain scree habitats are resource-poor, and this might be related to the dominance of sexual species as oribatid mites in other resource-poor habitats, such as tropical soils, dead wood, fungal sporocarps, salt marshes and caves, also are dominated by sexual species (Scheu and Drossel 2007; Maraun et al. 2019). By contrast, in habitats with ample resources and high densities of oribatid mites, such as boreal and temperate forests, parthenogenetic species dominate (Maraun et al. 2012).

### Species composition

All 17 species collected at the two study sites were Brachypylina. Many of the species are known from trees, rocks and caves. *Carabodes labyrinthicus* exclusively lives on lichens (Reeves 1988; Erdmann et al. 2007; Fischer et al. 2010) and stable isotope values suggest that this species also feeds on lichens. Ten species were assigned to primary decomposers (*C. marginatus*, *D. acutus*, *E. globosus*, *E. acromios*, *E. torulosus*, *H. gibba*, *L. coracinus*, *O. calcarata*, *P. willmanni*, *X. tegeocranus*) known to feed on detritus or fungi (Schneider et al. 2004; Norton and Behan-Pelletier 2009; Fischer et al. 2010; Maraun et al. 2011). Their presence indicates that in the scree habitat small amounts of organic material are present. Six species were assigned to secondary decomposers (*C. areolatus*, *C. bipilis*, *C. cuspidatus*, *O. foliata*, *O. quadricornuta*, *O. tibialis*). These species are also often found in forest soils but some of them are also regularly found in caves, namely *C. areolatus*, *C. bipilis* and *C. cuspidatus* (Bruckner 1995). Their high stable isotope signatures and their occurrence in resource-poor habitats such as caves indicates that these species are able to cope with extremely resource poor conditions and feed on the few resources present in mountain scree or caves (Mock et al. 2005). Very likely, their main food resources are fungi (Hågvar and Steen 2013; Hågvar et al. 2014). This is also supported by other stable isotope studies which found these species to be mainly secondary decomposers feeding on fungi (Fischer et al. 2010; Maraun et al. 2011; Erdmann et al. 2012; Bluhm et al. 2015; Maaß et al. 2015). Notably, the three *Oribatella* species (*O. quadricornuta*, *O. foliata* and *O. calcarata*) as well as the three species of the genus *Carabodes* (*C. areolatus*, *C. marginatus* and *C. labyrinthicus*) were ascribed to different trophic levels indicating that different species within genera may consume different resources.

Several oribatid mite species (e.g., *O. quadricornuta*, *O. calcarata*, *Parachipteria willmanni*) had high variation in ^15^N signatures indicating that their trophic niche is rather broad and that they feed on a wide range of resources. For example, *P. willmanni* occurs in forest soils, but also in peat bogs and in mountain scree (Wood and Lawton 1973; Lehmitz and Maraun 2016). This species may feed mainly on fungi, but it may also occasionally ingest resources such as mosses, which would explain their rather low ^15^N signatures and their high variation.

δ^13^C values were high for *L. coracinus*, *H. gibba*, *X. tegeocranus* and *D. acutus*. This was known before for *Liacarus* and *Carabodes* species (Maraun et al. 2011), and is likely due to the fact that these species incorporate calcium carbonate to harden their exoskeleton (Norton and Behan-Pelletier 1991). As the standard for ^13^C measurement (Pee Dee Belemnite limestone) with a ^13^C signature of zero reflects, inorganic carbonates are much less depleted in ^13^C than organic matter.

Overall, results of our study show that mountain scree habitats are colonized by specific oribatid mite communities comprising few species of derived taxa that reproduce sexually. Including lichen feeders, the species spanned three trophic levels, but predatory species were lacking suggesting limited resource availability for higher trophic levels. Low densities, sexual reproduction and few trophic levels all presumably reflect resource poor conditions in mountain scree habitats.
